# Response to Wang et al. and Shen et al.

**DOI:** 10.1093/jnci/djag189

**Published:** 2026-07-03

**Authors:** Ding Quan Ng, Julia Trudeau, Wen-Pin Chen, Argyrios Ziogas, Lifang Xie, Sanghoon Lee, Alexandre Chan

**Affiliations:** Department of Clinical Pharmacy Practice, School of Pharmacy, University of California Irvine, Irvine, CA, United States; Department of Clinical Pharmacy Practice, School of Pharmacy, University of California Irvine, Irvine, CA, United States; Biostatistics Shared Resource, Chao Family Comprehensive Cancer Center, Orange, CA, United States; Biostatistics Shared Resource, Chao Family Comprehensive Cancer Center, Orange, CA, United States; Susan Samueli Integrative Health Institute, University of California Irvine, Irvine, CA, United States; College of Korean Medicine, Kyung Hee University, Seoul, South Korea; Department of Clinical Pharmacy Practice, School of Pharmacy, University of California Irvine, Irvine, CA, United States

We thank Wang et al.^1^ and Shen et al.^2^ for their interest in our pilot randomized trial comparing the preliminary efficacy and safety of neuropsychiatric-specific electroacupuncture (nEA) with sham electroacupuncture (sEA) at alleviating cancer-related cognitive impairment (CRCI), fatigue, distress, and insomnia in breast cancer survivors.

Wang et al. highlighted the challenges in selecting an appropriate control group in acupuncture research. In our study, we intentionally designed a rigorous, active control to evaluate acupoint combinations that are effective for neuropsychiatric symptom clusters. Although the treatment acupoints (nEA) were selected based on both meridian theory and scientific evidence, the comparative acupoints (sEA) had relatively less modern scientific and clinical evidence despite having theoretical and traditional relevance. Further, the comparative acupoints TE8 and TE9 were also used in a sham control protocol for insomnia and depression.^3^ By comparing two acupoint combinations rather than using inert, non-acupoint controls, we have found targeted electroacupuncture, informed by scientific, clinical, and acupuncturist expert knowledge, represents a promising treatment for CRCI and related neuropsychiatric symptoms in breast cancer.

Shen et al. suggested that the treatment effect of acupoint selection should ideally be estimated via between-group primary comparisons. However, a lack of existing variance estimates for neuropsychiatric outcomes based on disease vs non-disease acupoint selection limited the feasibility of this comparison at this stage. To mitigate the risk of overestimating findings from within-group analyses, we applied the Benjamini-Hochberg procedure to reject false positive signals. Pre-post changes were also presented as Glass’s Δ to explicitly evaluate changes relative to baseline variance. Importantly, we emphasize that sample size calculations for a future phase III trial should be based on between-group differences and their associated variance estimates derived from this pilot.

Blinding outcomes are critical and should be consistently reported in acupuncture trials. With our findings of more correct guesses among nEA (50%) than sEA (31%) and less effective blinding in the nEA arm (Bang’s blinding indices^4^ = 0.357 [nEA] vs −0.062 [sEA]), we performed sensitivity analyses conditioning on treatment guesses as a robustness check against a conservative hypothesis that differential unblinding had inflated efficacy findings in the nEA group.^5^ We agree that adjusting for post-randomization covariates such as treatment guesses risks collider bias, whereby patients’ perception of efficacy may influence their guesses, introducing bias in an uncertain direction. Consequently, this is performed as a sensitivity analysis rather than the primary analysis.

Lastly, the full analysis of the EAST study, aligning with our published protocol, which includes our prespecified primary endpoint of between-group differences in self-perceived cognitive function, is planned for future publication. This current study reports data from the breast cancer subgroup of the EAST study, which is consistent with our protocol.^6^ Findings from another subgroup, comprising adolescent and young adult cancer patients, were reported separately at the 2026 ASCO Annual Meeting^7^ and are under review as a future publication. Reporting subgroup findings in stages is a common and accepted practice, particularly for pilot studies, and does not constitute misrepresentation. Ultimately, we reiterate that validation through a large, multicenter trial remains necessary.

## Data Availability

The datasets generated and/or analyzed during the current study are not publicly deposited but are available from the corresponding author on reasonable request.
